# Comprehensive splicing functional analysis of DNA variants of the *BRCA2 *gene by hybrid minigenes

**DOI:** 10.1186/bcr3202

**Published:** 2012-05-25

**Authors:** Alberto Acedo, David J Sanz, Mercedes Durán, Mar Infante, Lucía Pérez-Cabornero, Cristina Miner, Eladio A Velasco

**Affiliations:** 1 Grupo de Splicing y Cáncer, Instituto de Biología y Genética Molecular (IBGM), Consejo Superior de Investigaciones Científicas (CSIC)-Universidad de Valladolid, Sanz y Forés 3, Valladolid 47003, Spain; 2 Laboratorio de Cáncer Hereditario, Instituto de Biología y Genética Molecular (IBGM), Consejo Superior de Investigaciones Científicas (CSIC)-Universidad de Valladolid, Sanz y Forés 3, Valladolid 47003, Spain

## Abstract

**Introduction:**

The underlying pathogenic mechanism of a large fraction of DNA variants of disease-causing genes is the disruption of the splicing process. We aimed to investigate the effect on splicing of the *BRCA2 *variants c.8488-1G > A (exon 20) and c.9026_9030del (exon 23), as well as 41 *BRCA2 *variants reported in the Breast Cancer Information Core (BIC) mutation database.

**Methods:**

DNA variants were analyzed with the splicing prediction programs NNSPLICE and Human Splicing Finder. Functional analyses of candidate variants were performed by lymphocyte RT-PCR and/or hybrid minigene assays. Forty-one BIC variants of exons 19, 20, 23 and 24 were bioinformatically selected and generated by PCR-mutagenesis of the wild type minigenes.

**Results:**

Lymphocyte RT-PCR of c.8488-1G > A showed intron 19 retention and a 12-nucleotide deletion in exon 20, whereas c.9026_9030del did not show any splicing anomaly. Minigene analysis of c.8488-1G > A displayed the aforementioned aberrant isoforms but also exon 20 skipping. We further evaluated the splicing outcomes of 41 variants of four *BRCA2 *exons by minigene analysis. Eighteen variants presented splicing aberrations. Most variants (78.9%) disrupted the natural splice sites, whereas four altered putative enhancers/silencers and had a weak effect. Fluorescent RT-PCR of minigenes accurately detected 14 RNA isoforms generated by cryptic site usage, exon skipping and intron retention events. Fourteen variants showed total splicing disruptions and were predicted to truncate or eliminate essential domains of BRCA2.

**Conclusions:**

A relevant proportion of *BRCA2 *variants are correlated with splicing disruptions, indicating that RNA analysis is a valuable tool to assess the pathogenicity of a particular DNA change. The minigene system is a straightforward and robust approach to detect variants with an impact on splicing and contributes to a better knowledge of this gene expression step.

## Introduction

Germline mutations in the *BRCA1 *(MIM# 113705) and *BRCA2 *(MIM# 600185) genes confer a high lifetime risk of developing breast/ovarian cancer [1] and account for about 16% of the breast cancer familial risk [2]. Nearly 3,500 different DNA variants of *BRCA1 *and *BRCA2 *have been reported at the Breast Cancer Information Core Database (BIC) [3]. Only truncating mutations (1,457 nonsense and frameshift variants, 41.6%), variants in natural splice sites (141, 4%) and a minor fraction of the 1,346 BIC missense variants have been catalogued as deleterious [4,5]. Little is known about the pathogenicity of most of the remaining variants (approximately 50%), usually referred to as unclassified variants (UV), which complicates genetic counseling in UV carrier families [6]. A more reliable interpretation and classification of these sequence changes will improve the clinical management of cancer patients and their families. Several methods have been developed to classify them, including functional assays [7] and integrated statistical analysis of several parameters [8,9].

Genetic variants in disease-responsible genes that disrupt the splicing code have a key role in human hereditary disorders [10,11]. In fact, it has been estimated that more than 60% of sequence variations may affect pre-mRNA processing [12]. Therefore, splicing disruptions should be considered a core mechanism of gene inactivation to be investigated in UVs. Splicing mutations are traditionally considered those that affect essential nucleotides of the donor (GT) and the acceptor (AG) sites [13], although other intronic and exonic nucleotides are critical for splice site selection [14]. Furthermore, exon recognition is supported by other essential cis-regulatory motifs, the so-called splicing enhancers (ESE, ISE) or silencers (ISE, ISS) that are usually bound by SR and hnRNP proteins, respectively. They are short (6 to 8 nucleotides) and degenerate sequences that can promote (enhancers) or repress (silencers) exon inclusion in the mature mRNA. Sequence variations in any of these elements can result in alterations of the pre-mRNA processing step that can also be associated with human disorders, including hereditary cancer [14-18].

Identification of the splicing regulatory elements (SRE) by computational predictions is not accurate because of the degeneracy of these motifs [11,14,19,20], supporting the importance of RNA analysis to demonstrate the deleteriousness of a particular DNA change. However, it is often complex to obtain extra blood samples for RNA extraction from patients carrying a suspicious DNA change. Splicing functional analysis with hybrid minigenes is a simple and robust approach to study potential variants with impact on splicing without the need of collecting additional blood samples from patients. The genomic region of interest (exons and their flanking introns) from control and affected individuals can be cloned into a splicing reporter plasmid such as pSPL3. The construct is then transiently transfected into eukaryotic cells and the splicing pattern is analyzed by RT-PCR with specific primers of plasmid exons. The minigene assay constitutes a useful approach for identifying splicing anomalies and the study of their underlying functional mechanisms [21,22]. We previously studied 57 different pre-selected DNA variants from *BRCA1 *and *BRCA2 *using a combined approach: bioinformatics analysis and splicing assays by lymphocyte RNA RT-PCR and/or hybrid minigenes. Twenty-eight out of the 57 pre-selected variants displayed abnormal splicing patterns, suggesting that disruption of this process is an important disease mechanism [14].

In this study, we have performed a splicing analysis of *BRCA2 *variants c.8488-1G > A (acceptor site of exon 20) and c.9026_9030del (exon23) by lymphocyte RT-PCR and splicing reporter minigenes of exons 19-20 and 23-24. Moreover, we have extended this analysis to another 41 candidate splicing DNA variants of these exons reported in the BIC mutation database. A total of 19 variants induced aberrant splicing and we identified and quantified up to 12 different aberrant RNA isoforms from minigenes of exons 19-20 and 23-24.

## Materials and methods

### Patients, nucleic acid isolation and mutation detection

Breast and/or ovarian cancer patients harboring variants c.8488-1G > A and c.9026_9030del (nine families) were selected by the Genetic Counseling Unit (Complejo Hospitalario de Burgos) [23]. Written, informed consent was obtained from all patients prior to blood extraction. This study was approved by the ethics committee of the Faculty of Medicine, University of Valladolid (Spain). DNA and RNA were purified from peripheral blood lymphocytes of patients VA1220 (c.8488-1G > A) and VA1612 (c.9026_9030del) by using the QIAamp DNA and RNA blood mini kit (Qiagen, Hilden, Germany), respectively. RNA purification included a DNAse I treatment step. Mutation detection was performed by capillary heteroduplex analysis [24,25]. Nomenclature of sequence variations follows the guidelines of the Human Genome Variation Society (HGVS) [26], and was based on the *BRCA2 *cDNA sequence NM_000059.

### Splicing prediction programs

To identify potential variants with impact on splicing, mutant and reference sequences were analyzed with the following programs: a) NNSPLICE, which evaluates the strength of splice sites [27], and Human Splicing Finder (HSF) [28], which includes several matrices to analyze splice sites and splicing silencers and enhancers (for example, MaxEnt, ESEfinder, PESX and so on). In order to improve the specificity of ESEfinder predictions [29] we examined the evolutionary conservation of ESE motifs by aligning the *BRCA2 *sequences of different organisms with CLUSTALW2 [30].

### Lymphocyte RT-PCR

Lymphocyte RNA from patients carrying variants c.8488-1G > A (exon 20) and c.9026_9030del (exon 23) was retrotranscribed with the High Capacity kit (Applied Biosystems, Carlsbad, CA, USA). Amplification of cDNA was conducted with GoTaq Hot Start DNA polymerase (Promega, Madison, WI, USA) and flanking exonic primers forward RTBR2_EX19-FW, 5'AAACTTGGATTCTTTCCTGAC 3' (exon 19), and reverse RTBR2_EX23-RV, 5'ATTCTGTATCTCTTTCCTTCTGTT 3' (exon 23), for variant c.8488-1G > A (amplicon length: 653 bp), and RTBR2_EX22-FW, 5' GTGAAGAGCAGTTAAGAGCCT 3' (exon 22), and RTBR2_EX26-RV, 5' CTTGTTTTCTGCTTCATTGC 3' (exon 26) for variant c.9026_9030del (amplicon length: 773 bp). Splicing profiles of patients were compared with those obtained from RT-PCR experiments of control lymphocytes and non-tumorigenic breast epithelial cells (MCF10A; ATCC, LGC Standards) [31]. RT-PCR products were run on 1.5% agarose gels and stained with ethidium bromide. Isoforms induced by variant c.8488-1G > A were sequenced with the Big Dye 3.1 Sequencing Kit (Applied Biosystems) and primers RTBR2_EX19-FW and RTBR2_EX23-RV.

### Construction of minigenes

Wild type (wt) exons 19-20 (intron 18-exon 19-intron 19-exon 20-intron 20) and 23-24 (intron 22-exon23-intron 23-exon 24-intron 24) of *BRCA2 *were amplified with Pfx50 high fidelity polymerase (Invitrogen, Carlsbad, CA, USA) and primers with a restriction site (underlined) for either *Xho*I or *Bam*HI: **MGBR2_ex19-20_XhoI-FW**, 5' CACACACTCGAGATAGCATTAAGAACTTGTAGCA 3', and **MGBR2_ex19-20-BamHI-RV**, 5' CACACAGGATCCATTACAAATGGCTTAGACCTGA 3' (Exons 19-20; size: 1164 bp); and **MGBR2_ex23-24_XhoI-FW**, 5' CACACACTCGAGAATGCTTTGTTTTTATCAGTTTT 3', and **MGBR2_ex23-24_BamHI-RV**: 5' CACACAGGATCCAATTTGCCAACTGGTAGCTC 3' (exons 23-24; size: 717 bp).

After digestion with XhoI and BamHI (Fermentas, Vilnius, Lithuania), fragments were ligated to the exon trapping vector pSPL3 (Invitrogen, discontinued) to transform DH5α competent cells (Invitrogen). The new constructs were sequenced with the Big Dye 3.1 Sequencing Kit (Applied Biosystems) and primers PSPL3-SEQ-FW (5' CCTTGGGATGTTGATGAT 3') and PSPL3-SEQ-RV (5' TTGCTTCCTTCCACACAG 3').

### Site directed mutagenesis

Mutagenesis was carried out according to the PCR mutagenesis protocol with Pfu Turbo DNA polymerase (Agilent, Santa Clara, CA, USA) [32]. Wild type (wt) minigenes of *BRCA2 *exons 19-20 and 23-24 were used as templates to generate 41 candidate variants from the BIC database [see Additional file 1, Table S1]. In addition, several artificial variants were designed to target putative regulatory motifs: c.8484A > T and c.8486A > C, c.8512T > A, c.8518del and two combined BIC variants c.[8609A > G;8611G > T] and c.[8972G > A; 9006A > T] within the same construction. Finally, two conserved motifs of intron 19 were deleted separately and together: c.8487+31_8487+42del, c.8488-49_8488-44del, and c.[8487+31_8487+42del; c.8488-49_8488-44del].

### Transfection of HeLa cells

Approximately 10^5 ^HeLa cells (human cervical carcinoma) were grown to 90% to 95% confluency in 0.5 mL of growth medium ((D)MEM, 10% fetal bovine serum, 1% glucose and 1% penicillin/streptomycin) in four-well plates (Nunc, Roskilde, Denmark). Cells were transfected with 1 μg of plasmid minigene and 2 μL of lipofectamine 2000 (Invitrogen) following the manufacturer's protocol. After 48 hours RNA was purified with Nucleospin-RNA-II (Macherey-Nagel, Düren, Germany) that includes on-column rDNAse treatment and quantified in a Nanodrop 1000 spectrophotometer (Fisher Scientific, Spain).

### RT-PCR of minigenes and quantification of mRNA isoforms

Retrotranscription was carried out with 200 ng of RNA and the transcriptor first strand cDNA synthesis kit (Roche, Sant Cugat del Vallés, Barcelona, Spain). Semiquantitative fluorescent RT-PCRs were performed in triplicate in a final volume of 20 μL that contained 2 μL of cDNA and the flanking primers of constitutive exons of pSPL3, SD6-PSPL3_RTFW (FAM-5' TCACCTGGACAACCTCAAAG 3') and SA2-PSPL3_RTREV (5' TGAGGAGTGAATTGGTCGAA 3'). Samples were denatured at 94°C for five minutes, followed by 26 cycles consisting of 94°C for 20 seconds, 58°C for 20 seconds, and 72°C for 30 seconds, and a final extension step at 72°C for two minutes. Sizes of the RT-PCR products were 486 and 488 bp for minigenes 19-20 and 23-24, respectively. The RT-PCR products (1 μL of a 1/10 dilution) were mixed with 18 μL of Hi-Di Formamide (Applied Biosystems) and 0.2 μL of Genescan 500 Rox Size Standard (Applied Biosystems). Samples were run on an ABI3130 sequencer using POP7 polymer and analyzed with the Peak Scanner software (Applied Biosystems). Mean peak areas were used to calculate ratios of the different splicing isoforms generated by DNA variants from minigenes 19-20 and 23-24.

Sequencing of the minigene RT-PCR products was carried out as described above with primers SD6-PSPL3_RTFW and SA2-PSPL3_RTREV except for intron 19 retention isoform that was sequenced with a specific primer in the boundary intron 19-exon 20 (RTBR2_ivs19-ex20-RV: 5' TGTCTTCTCCATCCACTGTAAT 3').

## Results and discussion

**Figure 1:**
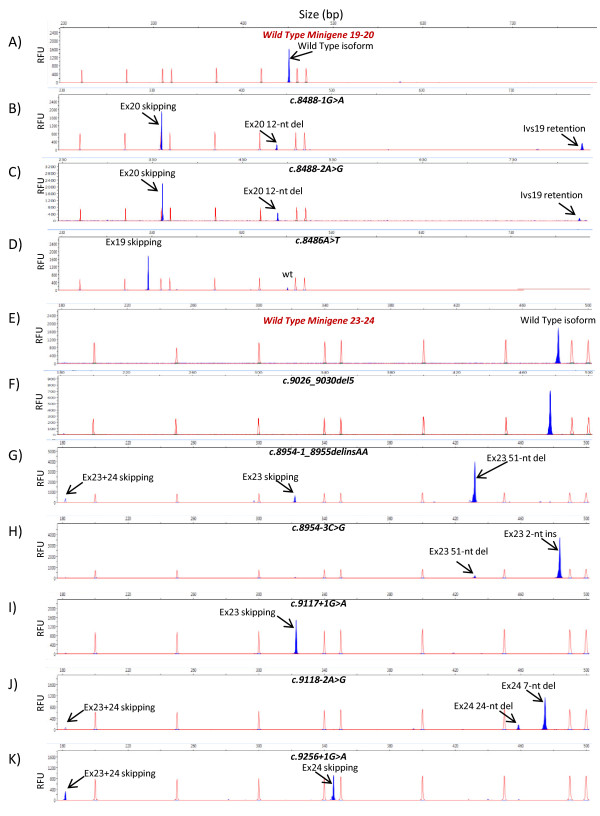
**Splicing outcomes of variants of exons 19-20 (A-D) and 23-24 (E-K) of *BRCA2***. RT-PCRs labelled with FAM (shadowed blue peaks) of mutant minigenes were run in an ABI3130 sequencer with Genescan ROX 500 (red peaks) as size standard. Aberrant isoforms are indicated by arrows. RFU: Relative Fluorescence Units. **A-D**) Chromatograms of the wt minigene and variants of c.8488-1G > A (intron 19), c.8488-2A > G (intron 19), and c.8486A > T (exon19). **E-K**) Chromatograms of the wt minigene and variants c.9026_9030del (exon 23), c.8954-1_8955delinsAA (intron 22-exon23), c.8954-3C > G (intron 22), c.9117+1G > A (intron 23), c.9118-2A > G (intron 23), and c.9256+1G > A (intron 24). wt, wild type.

Variant c.8488-1G > A was detected in a patient who developed bilateral breast cancer at age 66 and 74 and ovarian cancer at age 77. This variant affects the essential nucleotide -1 (G to A) of the acceptor splice site of *BRCA2 *exon 20. This variant had not been reported at the BIC mutation database although it had formerly been found in a consanguineous Fanconi anemia patient [33]. Analysis with HSF at the intron/exon boundary identified a weak acceptor splice site that was even weakened by the change -1 G > A. Lymphocyte RT-PCR of the carrier patient revealed an upper band with intron 19 retention (1053 bp) and a transcript with a 12-nucleotide deletion of exon 20 generated by the use of an alternative cryptic acceptor [see Additional file 1, Figure S1]. This variant was also evaluated by hybrid minigenes of exons 19-20. Splicing assay and fluorescent RT-PCR (Figure 1B and Additional file 1, Figure S1) revealed three main isoforms: 12-nucleotide deletion of exon 20 (9.7%), intron 19 retention (16.0%), exon 20 skipping (72.1%), which was not previously identified in lymphocytes, and other minor isoforms. The different splicing profiles between both assays may be due to the different genomic context where adjacent exons may be involved in the splicing efficiency of exons 19 and 20 [34]. The most common events of alternative splicing are exon skipping and alternative splice site selection whereas intron retention is the less frequent phenomenon in physiological alternative splicing [35], indicating that its contribution to the complexity of the human proteome is low since this event is usually associated with the introduction of premature stop codons [36]. Moreover, intron retention is increased by two-fold in cancerous cells, suggesting the disruption of essential repressor genes of tumor progression [36]. Nevertheless, intron retention rate can be particularly high in some specific genes such as the Kallikrein gene family of serine proteases, in which six isoforms showed intron III exonization and were predicted to truncate all the resultant proteins [37].

Translation of the intron 19 retention and exon 20 skipping isoforms was predicted to result in truncated proteins (p.Gln2829fsX2 and p.Trp2830LysfsX13, respectively), whereas the 12-nucleotide deletion produced an in frame deletion of four amino acids (p.Trp2830_Lys2833del, W-M-E-K) of the *BRCA2 *DNA binding domain. Tryptophan 2830 and glutamic acid 2832 are strictly conserved in vertebrates, (IARC *BRCA2 *alignment), suggesting their functional relevance [38]. Moreover, one Fanconi anemia patient of subtype D1 (associated with *BRCA2 *mutations) with a family history of consanguinity was previously reported to be homozygous for this variant [33]. Altogether these data strongly support the pathogenicity of c.8488-1G > A.

*BRCA2 *c.9026_9030del of exon 23 is a deleterious frameshift mutation that would lead to a premature stop codon (p.Tyr3009SerfsX9). It is one of the most prevalent mutations in the Spanish population, accounting for 7.6% of *BRCA2 *families in Castilla y León (Spain) [39]. We had already shown that other deletions, such as *BRCA2 *c.470_474del, can induce splicing defects [14]. HSF analysis predicted changes in several presumed regulatory elements (Exon and Intron Identity Elements) [40]. We performed lymphocyte RT-PCR of one carrier patient and we found the wt allele and the corresponding 5-nucleotide deletion transcript derived from the genomic variant (c.9026_9030del) without any splicing anomaly (data not shown). This result was confirmed in the minigene of exons 23 and 24 (Figure 1F).

### Minigene analysis of BIC variants

We had previously shown that first, more than 20% of *BRCA *variants reported at the BIC database can induce splicing disruptions and, second, the combination of PCR mutagenesis and splicing reporter plasmids is a straightforward and strong approach that allows the analysis of the splicing effect of any sequence change [14]. We, therefore, proceeded to analyze all the reported variants of exons 19, 20, 23 and 24 from the BIC database (155 different DNA changes) with NNSplice and HSF. We chose variants that met one of the following criteria: disruption of the natural splice site, creation of novel alternative donor or acceptor sites, alteration of evolutionarily conserved ESEs, and creation of splicing silencers. A total of 41 variants (26.5%) were preselected and generated by PCR mutagenesis in the wt minigenes 19-20 and 23-24. Splicing functional assays were carried out by semiquantitative fluorescent RT-PCR and the peak areas of the different isoforms were quantified. Eighteen variants (43.9%) produced aberrant splicing patterns (Table 1, Figure 1 and Additional file 1, Figure S2) that affected the natural splice sites (12 variants: c.8487+1G > A, c.8487+3A > G, c.8488-2A > G, c.8954-3C > G, c.8954-1_8955delinsAA, c.9117G > A, c.9117+1G > T, c.9117+1G > A, c.9118-2A > G, c.9248_9256+7del, c.9256G > T and c.9256+1G > A), splicing enhancers or silencers (four variants: c.8378G > A, c.8969G > A, c.9006A > T and c.9076C > T) or both of them simultaneously, splice site and enhancer (c.8486A > T and c.8487G > A). Interestingly, variant c.8488-2A > G (Figure 1C) replicated the splicing pattern of c.8488-1G > A (Figure 1B) although slight differences in the relative proportions of each isoform were observed [see Additional file 1, Figure S3]. Variants c.8486A > T and c.8487G > A of the penultimate and last nucleotides of exon 19, respectively, decreased the canonical donor splice site score of intron 19 (MaxEnt -480% and -502%, respectively) and NNSPLICE (0.95→0.87 and 0.95→0.4, respectively) but also disrupted one putative SF2/ASF enhancer. To ascertain the nature of these splicing anomalies two artificial variants were designed and generated: c.8484A > T (intact splice site and SF2 disruption) and c.8486A > C (weak alteration of splice site: 0.95 →0.92 and SF2 disruption; Additional file 1, Table S1). The first one, c.8484A > T, only revealed the wt isoform whereas c.8486A > C produced total exon 19 skipping [Additional file 1, Figure S3]. Consequently, only those variants that were predicted to affect the donor site, even slightly (c.8486A > T, c.8486A > C and c.8487G > A), altered the splicing process, suggesting that this is the causative mechanism. Actually, the last and penultimate exonic nucleotides of exons as well as the intronic positions +1 to +5 and -3 to -1 are also highly conserved [11] and should be considered potential targets of variants with impact on splicing [41]. Finally, we also generated another five artificial variants, c.8512T > A and c.8518del (disruption of conserved SC35 and SRp55 ESEs), and three single and combined deletions of two conserved intron 19 motifs (c.8487+31_8487+42del, c.8488-49_8488-44del and c.[8487+31_8487+42del; c.8488-49_8488-44del]; Additional file 1, Table S1) that were genetically engineered in the respective minigenes but neither of them showed patent splicing anomalies.

**Table 1:** Bioinformatics analysis and RNA results of variants with impact on splicing of *BRCA2 *exons 19, 20, 23 and 24.

Mutation (HGVS)^a^	Bioinformatics analysis^c^	Splicing outcome^d^	RNA effect (HGVS)^a^	Predicted protein effect (HGVS)^a^
**Exons 19-20^b ^**		WT Minigene 19-20: N_Sp(99%)		

c.8378G > A (ex19)	[-] ESE, [+] hnRNP-B	*N_Sp/Ex19 skipping *	r.[8378g > a, 8332_8487del]	p.[G2793E, Ile2778_Gln2829del]
c.8486A > T (ex19)	↓score donor site/[-] SF2/ASF	Ex19 skipping	r.8332_8487del	p.Ile2778_Gln2829del
c.8487G > A (ex19)	↓score donor site/[-] SF2/ASF	Ex19 skipping	r.8332_8487del	p.Ile2778_Gln2829del
c.8487+1G > A (ivs19)	[-] Donor site disruption.	Ex19 skipping	r.8332_8487del	p.Ile2778_Gln2829del
c.8487+3A > G (ivs19)	↓score donor site.	Ex19 skipping	r.8332_8487del	p.Ile2778_Gln2829del
c.8488-2A > G (ivs19)	[-] Acceptor site	Intron retention/Ex20_12nt deletion/Ex20 skipping	r.[8487_8488ins8487+1_8488-1, 8488_8499del, r.8488_8632del]	p.[Gln2829fsX2, Trp2830_Lys2833del Trp2830LysfsX13]
c.8488-1G > A (ivs19)	[-] Acceptor site	Intron retention/Ex20_12nt deletion/Ex20 skipping	r.[8487_8488ins8487+1_8488-1, 8488_8499del, r.8488_8632del]	p.[Gln2829fsX2, Trp2830_Lys2833del Trp2830LysfsX13]

**Exons 23-24^b^**		WT Minigene 23-24: N_Sp (100%)		

c.8954-3C > G (ivs22)	[+] Acceptor site 2-nt upstream	Ex23_2-nt insertion	r.8954-2_8954-1ins	p.Val2985GlufsX4
c.8954-1_8955delinsAA (ivs22-ex23)	Disruption acceptor site	Ex23_51-nt deletion/Ex23 skipping/Ex23+24 skipping	r.[8954_9004del, 8954_9117del, 8954_9256del]	p.[Val2985_Thr3001del, Val2985GlyfsX3, p.Val2985_Thr3085del]
c.8969G > A (ex23)	[+] hnRNPA1; [+] hnRNP-B	*N_Sp/Ex23_51-nt del *	r.[8969g > a, 8954_9004del]	p.[W2990X, Val2985_Thr3001del]
c.9006A > T (ex23)	[-] SRp40; ↑score cryptic site	*N_Sp/Ex23_51-nt del *	r.[9006a > u, 8954_9004del]	p.[E3002D, Val2985_Thr3001del]
c.9076C > T (ex23)	[+] ESS	*N_Sp/Ex23 skipping/Ex23+24 skipping *	r.[9076c > u, 8954_9117del, 8954_9256del]	p.[Q3026X, Val2985GlyfsX3, Val2985_Thr3085del]
c.9117G > A (ex23)	[-] Donor site	Ex23 skipping/Ex23_51-nt del/Ex23+24 skipping	r.[8954_9117del, 8954_9004del, 8954_9256del]	p.[Val2985GlyfsX3, Val2985_Thr3001del, Val2985_Thr3085del]
c.9117+1G > T (ivs23)	[-] Donor site	Ex23 skipping	r.8954_9117del	p.Val2985GlyfsX3
c.9117+1G > A (ivs23)	[-] Donor site	Ex23 skipping	r.8954_9117del	p.Val2985GlyfsX3
c.9118-2A > G (ivs23)	[-] Acceptor site. Novel acceptor 7-nt downstream	Ex24_7-nt del/Ex24_24-nt del/Ex23+24 skipping	r.[9118_9124del, 9118_9141del, 8954_9256del]	p.[Val3040MetfsX20, Val3040_Gln3047del, Val2985_Thr3085del]
c.9248_9256+7del (ex24-ivs24)	[-] Donor site	Ex24 skipping/Ex24_43-nt del/Ex23+24 skipping	r.[9118_9256del, 9214_9256del, 8954_9256del]	p.[Val3040AspfsX18, Val3072AspfsX18, Val2985_Thr3085del
c.9256G > T (ex24)	[-] Donor site	*Ex24 skipping/Ex23+24 skipping/Ex24_43-nt del/N_Sp *	r.[9118_9256del, 8954_9256del, 9214_9256del,9256g > u]	p.[Val3040AspfsX18, Val2985_Thr3085del, Val3072AspfsX18, G3086X]
c.9256+1G > A (ivs24)	[-] Donor site	Ex24 skipping/Ex23+24 skipping/Ex24_43-nt del	r.[9118_9256del, 8954_9256del, 9214_9256del]	p.[Val3040AspfsX18, Val2985_Thr3085del, Val3072AspfsX18]

^a^Nomenclature of variants at DNA, RNA and protein levels follows the guidelines of the Human Genome Variation Society. ^b^Intronic (ivs) or/and exonic (ex) location of each variant are indicated between brackets. ^c^Summary of bioinformatics results with NNSPLICE and Human Splicing Finder. [-], disruption; [+], creation. ESE, Exonic Splicing Enhancer; ESS, Exonic Splicing Silencer; SF2/ASF and SRp40 are SR proteins that bind ESE/enhancer motifs; hnRNP proteins bind ESS/silencer motifs. ^d^Major isoforms are described. EX, exon; nt, nucleotide; del, deletion. Wild type minigene of exons 19 and 20 produced a minimal amount (1%) of exon 19 and exons 19+20 skipping. Variants with a relevant fraction of the wild type isoform are italicized (N_Sp: Normal Splicing). The relative proportions of each isoform are shown in Additional file 1, Figure S3.

### Aberrant RNA isoforms and predicted effect on protein

**Figure 2:**
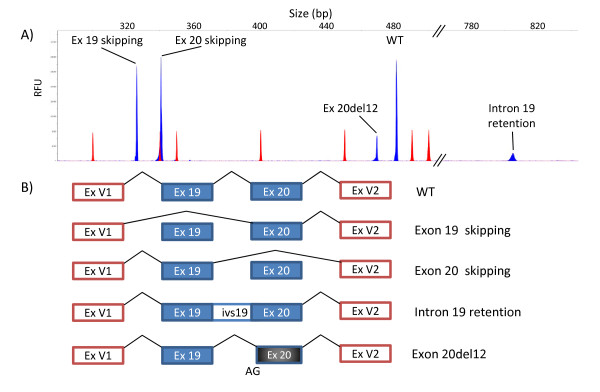
**Splicing isoforms generated by DNA variants of exons 19 and 20**. A) Fluorescent RT-PCRs (blue peaks) of mutant minigenes were run in an ABI3130 DNA sequencer with Genescan ROX 500 (red peaks) as size standard. Chromatograms of different variants were overlaid to generate this picture. **B**) Diagrams of the splicing outcomes caused by variants of exons 19 and 20.

**Figure 3:**
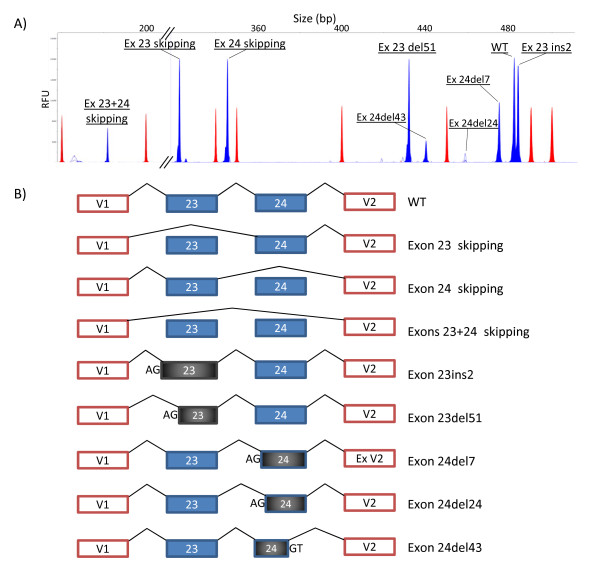
**Splicing isoforms generated by DNA variants of exons 23 and 24**. A) Fluorescent RT-PCRs (blue peaks) of mutant minigenes were run in an ABI3130 DNA sequencer with Genescan ROX 500 (red peaks) as size standard. Chromatograms of different variants were overlaid to generate this picture. **B**) Diagrams of the splicing outcomes caused by variants of exons 23 and 24.

A total of 14 different RNA isoforms, including the canonical transcripts, were detected in the minigene experiments of exons 19-20 (five isoforms) and 23-24 (nine isoforms; Additional file 1, Figure S2). The 12 aberrant isoforms (Figures 2 and 3) were caused by four different events including exon skipping (four isoforms: exons 19, 20, 23 and 24 skipping), double exon skipping (exons 23+24), intron 19 retention and use of alternative cryptic acceptor or donor splice sites (exon 20del12, exon 23ins2, exon 23del51, exon 24del7, exon 24del24 and exon 24del43). Moreover, most variants (12/19; Table 1) produced two or more distinct RNA isoforms. Likewise, the high resolution of the fluorescent RT-PCR technique in splicing reporter assays should be highlighted as it allowed accurate detection of isoforms that differed in size by as little as two (exon 23ins2) or seven nucleotides (exon 24del7) (Figure 3), which otherwise could be masked by the wt isoform in agarose gel electrophoresis [42]. Furthermore, its high resolution is capable of detecting minor transcripts (less than 5% of total mRNA isoforms; Additional file 1, Figure S3) that could not be visualized in agarose gels. Finally, the minigene assay is a single-allele assay that allows a precise quantification of the different RNA isoforms without the interference of the normal allele as in lymphocyte RT-PCR. However, the Nonsense-Mediated mRNA Decay (NMD) mechanism selectively degrades mRNAs harboring premature termination codons (PTC) that can impair the relative proportions of each isoform. In fact, 13 out of 14 transcripts, including the wt ones (Figures 2 and 3), do not keep the ORF of exon 1 of pSPL3 and are, therefore, susceptible to undergo NMD unless this process is inhibited in cell culture [43], so special care should be taken in interpreting these results.

With regard to the putative pathogenicity of variants with an effect on splicing, two basic criteria are considered biological indicators [16-18]: first, total or almost total splicing disruption (absence of the canonical transcript); and second, the predicted effects of aberrant transcripts on protein translation, such as introduction of frameshifts and premature stop codons or loss of essential domains of *BRCA2 *(Table 1). Thirteen BIC variants of the splice sites (c.8486A > T, c.8487G > A, c.8487+1G > A, c.8487+3A > G, c.8488-2A > G, c.8954-3C > G, c.8954-1_8955delinsAA, c.9117G > A, c.9117+1G > T, c.9117+1G > A, c.9118-2A > G, c.9248_9256+7del and c.9256+1G > A) induced major splicing defects that consisted of exon skipping, double exon skipping, insertion of nucleotides and partial deletions of exons due to the use of cryptic acceptor or donor sites. Translation of the anomalous transcripts was predicted to result in protein truncation (exon 20 skipping, intron 19 retention, exon 23 skipping, exon 24 skipping, exon 23ins2, exon 24del7 and exon 24del43) or in-frame deletions (ex19 skipping, exon 20del12, exon 23del51, exons 23+24 skipping, exon 24del24) of conserved amino acids of the essential BRCA2 DNA binding domain (Table 1) which spans amino acids 2500 to 3098, suggesting that these variants might have a role in the disease. On the other hand, variant c.9256G > T of exon 24 produced a significant fraction of the canonical transcript (42.3%; Additional file 1, Figure S3). However, the canonical transcript contains the nonsense change r.9256g > u, thus supporting a double deleterious mechanism: protein truncation (p.G3086X) and splicing disruption (three aberrant isoforms; Table 1). Other variants, such as *BRCA1 *c.5123C > A and *BRCA2 *c.8168A > G (both missense changes), had already been shown to alter protein function and pre-mRNA processing [4,5,14,44].

In contrast, the four SRE variants (ESE disruption and/or ESS creation; Table 1) had low to moderate effects on splicing since aberrant isoforms accounted for 8% (c.8969G > A) to 22% (c.9006A > T) of total transcripts. Up to now we have detected 16 SRE variants (two of them artificial variants) [14] of which five are placed in *BRCA2 *exon 5 suggesting that regulation of some exons strongly depends on supplementary cis-regulatory elements such as ESE or ESS. Thus, it is expected that each exon is regulated by different mechanisms that can only be determined experimentally. Another two putative silencer variants (c.9148C > T and c.9227G > T) showed minor splicing defects (< 2% of transcripts; data not shown) that were not considered relevant. Interestingly, the combination of two putative ESE variants, c.8972G > A (SRp55) and c.9006A > T (SRp40), in the same exon 23-24 minigene induced a greater splicing defect than each variant alone. In addition, variant c.9006A > T also strengthened the cryptic acceptor site 51 nucleotides downstream of the canonical one that is used in one aberrant isoform (exon 23del51; Figure 3). In single assays, c.8972G > A had no effect on splicing as previously reported [45] whereas c.9006A > T produced the canonical transcript (68.8%), exon 23del51 (19.7%), exon 23 skipping (9.2%) and exons 23+24 skipping (2.3%). Surprisingly, we found that the combination of both variants increased the fraction of the 51-nucleotide deletion isoform from 19.7% to 39.9% whereas the other anomalous isoforms decreased [See Additional file 1, Figures S3 and S4]. Furthermore, acceptor and donor sites of exon 23 are weak, suggesting that supplementary control elements are required for correct exon 23 recognition [46]. Altogether these data suggest that exon 23 recognition depends, at least in part, on the splicing factors SRp55 and SRp40 that might act cooperatively although this issue should be confirmed by RNA binding assays. Conversely, other SRE variants were previously proven to trigger complete splicing defects, such as *BRCA1 *c.5080G > T and *BRCA2 *c.93G > T, c.145G > T or c.470_474del [14,47].

Pathogenicity of variants with incomplete splicing outcomes is uncertain but they might constitute low-moderate penetrance alleles of breast/ovarian cancer as it occurs in CFTR-related disorders [48]. Thus, the penetrance and severity of this group of diseases are correlated with an abnormal number of UG and U repeats located in the acceptor site of exon 9 of the CFTR gene, which increase the exon 9 skipping rate. Consequently, genetic variation can affect splicing efficiency that can modify the severity of the disease phenotype or be linked with disease susceptibility [10]. In any case, additional epidemiological studies should be conducted to estimate accurately the breast/ovarian cancer risks of DNA variants with total and partial defective splicing outcomes.

## Conclusions

Taking all these results together, 12.2% of all the variants (19/155) of *BRCA2 *exons 19, 20, 23 and 24 are associated with splicing defects. They comprise three nonsense, three missense, two synonymous and 11 splice site variants (including two deletions), indicating that any DNA change can disrupt pre-mRNA processing. Furthermore, in this and previous reports [14], we have analyzed in depth 14 exons of *BRCA1 *and *BRCA2 *(541 different BIC DNA variants) and detected 45 natural variants with an impact on splicing of which 21 altered splice sites, 14 modified SREs (enhancer disruption and/or silencer creation), eight created alternative sites and two affected the polypyrimidine tract. However, taking into account all the critical positions of the splice sites (intronic -3 to -1 and +1 to +5, and the conserved exonic nucleotides) of those 14 exons, the number of variants with impact on splicing would therefore increase to 85 (15.7% of all reported BIC variants) that would account for 33.9% of all presumed deleterious mutations (113 frameshift, 53 nonsense and 85 splicing variants), more than two-fold higher than the classical estimate [13].

Computational predictions of variants at the splice sites were precise by the three algorithms used in this work, but only HSF was able to identify all the cryptic alternative sites of exons 20, 23 and 24 (Figures 2 and 3). In contrast, bioinformatics evaluations of enhancer inactivation or silencer creation were not so accurate and had a high false-positive rate. A complete knowledge of the splicing code will enhance sensitivity of these bioinformatics predictions [46]. However, the construction of one single prediction model seems to be an arduous task that will require comprehensive experimental validations [19].

Direct analysis of splicing anomalies in patient RNA should be the method of choice to identify variants with an effect on splicing but this approach presents two principal limitations. First, the patient sample might not be available and, second, leukocytes are the main source of RNA, so caution should be taken when interpreting these results since differential tissue-specific alternative splicing events could mask the real splicing outcome of a DNA variant [21,34]. In this context, splicing reporter minigenes are valuable tools to corroborate bioinformatics data. This and previous reports have also shown its importance for the identification of variants with an effect on splicing without the need of patient RNA samples that are usually difficult to obtain [14,18].

As a general rule, splicing profiles of patient and minigene RNA are very similar [14,19,49]. However, we have observed discrepancies between splicing anomalies of patient RNA and minigene assays, such as variants c.8488-1G > A (this work) and c.212+1G > A of *BRCA1 *exon 5 [14]. The clinical relevance of c.8488-1G > A has been discussed above and that of c.212+1G > A is strongly supported by family, bioinformatics (donor site disruption) and functional data as well as by the fact that other variants of the donor site of exon5/intron 5 (c.211A > G and c.212+3A > G) were definitely classified as deleterious mutations by several approaches [19,50,51]. Therefore, it seems that the observed differences may probably be due to a technical issue of minigene constructs with particular exons where the lack of the natural genomic environment without its neighboring exons can be responsible for the different results obtained. Moreover, it was previously reported that a minigene containing only exon 37 of the NF1 gene with the pathogenic variant 6792C > G principally induced exon 37 skipping. Conversely, a minigene with exons 31 to 38 with the same variant replicated almost exactly the splicing pattern of patient lymphoblasts (canonical transcript, exon 37 skipping and exons 36+37 skipping) [34]. Consequently, larger minigene constructions with more exons should be carried out in order to mimic the natural genomic background. The minigene system is a straightforward and robust assay that helps to classify DNA variants of unknown clinical significance under the splicing viewpoint, although these tests require further validation. As more data are collected, it will provide a more accurate risk estimation of breast and ovarian cancer associated with splicing alterations. Finally, analysis of minigene RT-PCR products in a DNA sequencer provides higher resolution than agarose electrophoresis [42,52], since we have shown that a precise identification of minor or rare transcripts and quantification of all the isoforms generated by a specific DNA change is possible (Figures 2 and 3).

In conclusion, an important fraction of DNA variants are associated with splicing aberrations that should be considered as a primary mechanism of gene inactivation to be investigated in unclassified DNA variants. These studies provide insights into the basic regulatory mechanisms of this step of eukaryotic gene expression contributing to a better knowledge of the rules for exon definition. Hence, splicing functional assays supply essential information to distinguish between neutral variants and variants with an impact on splicing and should be incorporated in genotype screenings of human hereditary diseases.

## Abbreviations

BIC: The Breast Cancer Information Core; ESE: exonic splicing enhancer; ESS: exonic splicing silencer; FAM: 6-carboxyfluorescein; HGVS: Human Genome Variation Society; hnRNP: heterogeneous nuclear ribonucleoproteins; ISE: intronic splicing enhancer; ISS: Intronic splicing silencer; NMD: nonsense-mediated mRNA decay; ROX: 6-carboxy-X-rhodamine; SR proteins: serine/arginine-rich proteins; SRE: splicing regulatory elements; UV: unclassified variant; WT: wild type.

## Competing interests

The authors declare that they have no competing interests.

## Authors' contributions

AA and DJS participated in the experimental design and bioinformatics analysis, and performed the splicing functional assays. MI and LP-C participated in the functional assays and bioinformatics analyses. MD and CM provided the breast/ovarian cancer patients included in this study as well as mutation data of patients from Castilla y León (Spain). EAV designed the study, conducted the bioinformatics analysis, supervised minigene construction and data analysis, and wrote the manuscript. All authors read and approved the final manuscript.

## Supplementary Material

Additional file 1 **Bioinformatics and RNA analyses of candidate DNA variants**. Bioinformatics analysis of putative splicing variants of exons 19, 20, 23 and 24 of BRCA2 (Table S1). Splicing outcomes of DNA variant c.8488-1G > A (Figure S1). Sequence chromatograms of the main splicing outcomes of the minigenes 19-20 and 23-24 of BRCA2 (Figure S2). Quantification of RNA isoforms induced by variants of minigenes of exons 19-20 and 23-24 (Figure S3). Cumulative effect of ESE mutations from BRCA2 exon 23 on splicing (Figure S4). Click here for file
