# Supplementary material for: Comprehensive splicing functional analysis of DNA variants of the BRCA2 gene by hybrid minigenes
Source: Breast Cancer Res. 2012 May 25;14(3):R87. doi: 10.1186/bcr3202 (PMC3446350; doi:10.1186/bcr3202)
Supplement: Additional file 1 — Bioinformatics and RNA analyses of candidate DNA variants. Bioinformatics analysis of putative splicing variants of exons 19, 20, 23 and 24 of BRCA2 (Table S1). Splicing outcomes of DNA variant c.8488-1G > A (Figure S1). Sequence chromatograms of the main splicing outcomes of the minigenes 19-20 and 23-24 of BRCA2 (Figure S2). Quantification of RNA isoforms induced by variants of minigenes of exons 19-20 and 23-24 (Figure S3). Cumulative effect of ESE mutations from BRCA2 exon 23 on splicing (Figure S4). [file bcr3202-S1.PDF]

Table S1. Bioinformatics analysis of putative splicing variants of exons 19, 20, 23 and 24 of *BRCA2*.

Mutation (HGVS nomenclature)	Source	Type of mutation	SUMMARY OF BIOINFORMATICS ANALYSIS
		EXONS 19-20	
c.8362T>C	BIC	Missense	[-] SC35, SRp40
c.8368A>T	BIC	Missense	[-] SC35
c.8378G>A	BIC	Missense	[-] ESE, [+] ESS (hnRNP-B)
c.8394_8396delTAGinsAA	BIC	Frameshift	[+] ESS
c.8398C>T	BIC	Missense	[-] SRp40, [+] ESS (hnRNP-I)
c.8435G>A	BIC	Missense	[-] SF2/ASF, [+] ESEs , [-] ESS
c.8438G>A	BIC	Missense	[+] 1 ESE, [+] 1 ESS
c.8484A>T	*Artificial*	*Synonymous*	*Donor site(exon 19): 0.95 → 0.95. // [-] SF2/ASF (ESEfinder)*
c.8486A>C	*Artificial*	*Missense*	*Donor site (exon 19): 0.95 → 0.92 (NNSPLICE) // [-] conserved SF2/ASF*
c.8486A>T	BIC	Missense	Donor site (exon 19): 0.95 → 0.87 (NNSPLICE) // [-] conserved SF2/ASF
c.8487G>A	BIC	Synonymous	Donor site (exon 19): 0.95→0.40 (NNSPLICE) // [-]conserved SF2/ASF
c.8487+1G>A	BIC	Splice Site	[-] Donor site (exon 19)
c.8487+3A>G	BIC	Splice Site	Donor site (exon 19): 0.95→0.78 (NNSPLICE). 44% decrease MaxENt score
c.8487+31_8487+42del	*Artificial*	*Intronic*	*del 12-nt conserved motif. Putative binding site of hnRNP-I*
c.8488-49_8488-44del	*Artificial*	*Intronic*	*del TGAATG motif: Intron Identity Element (IIE): silencer.*
c.[8487+31_8487+42del; c.8488-49_8488-44del] Combined	*Artificial*	*Intronic*	*del12-nt + del TGAATG*
c.8488-2A>G	BIC	Splice Site	[-] Acceptor site (exon 20) (Human Splicing Finder)
c.8488-1G>A	Patient	Splice Site	[-] Acceptor site (exon 20) (Human Splicing Finder)
c.8512T>A	*Artificial*	*Missense*	*[-] conserved SC35 motif*
c.8518del	*Artificial*	*Frameshift*	*[-] conserved SRp55 motif*
c.8539G>A	BIC	Missense	[-] conserved SF/ASF and SRp40 motifs
c.8560del	BIC	Frameshift	Creation of two silencers
c.8572C>A	BIC	Missense	[+] ESE, [+] ESS
c.[8609A>G;8611G>T] Combined	*Artificial*	-	*[-] conserved SRp40 motif*
		EXONS 23-24	
c.8954-3C>G	BIC	Splice Site	[-] Natural acceptor site(exon 23), [+] cryptic acceptor site 2-nt upstream
c.8954-1_8955delinsAA	BIC	Splice Site	[-] Acceptor site (exon 23)
c.8961_8964del	BIC	Frameshift	Acceptor (exon 23): 0.53→0.26 [-]SF2/ASF +SRp55, [+] ESS (PESX)
c.8969G>A	BIC	Nonsense	[+] hnRNPA1 (HSF), [+] hnRNP-B (ESR)
c.8972G>A	BIC	Missense	[-] SC35

**c.9004G>A**	**BIC**	**Missense**	**[-] SF2/ASF partially conserved and multiple SREs**
**c.9006A>T**	**BIC**	**Missense**	**[-] Conserved SRp40**
c.[8972G>A; 9006A>T] Combined	*Artificial*	-	*[-] SC35; [-] SRp40 conserved. ↑cryptic acceptor site.*
**c.9026_9030del**	**BIC/patients**	**Frameshift**	**[-] 2 Exon Identity Elements / [+] 2 Intron Identity Elements**
**c.9076C>T**	**BIC**	**Nonsense**	**Silencer creation (Rescue-ESE & PESX)**
**c.9100C>T**	**BIC**	**Nonsense**	**[-]SF2/ASF, [-] 1ESE / [+] 3 ESS, [+] hnRNPA1**
**c.9101A>G**	**BIC**	**Missense**	**[-] SF2/ASF (mammalian)**
**c.9116C>T**	**BIC**	**Missense**	**Donor site: 0.57→0.37**
**c.9117G>A**	**BIC**	**Synonymous**	**[-] Donor site (exon 23)**
**c.9117+1G>T**	**BIC**	**Splice Site**	**[-] Donor site (exon 23)**
**c.9117+1G>A**	**BIC**	**Splice Site**	**[-] Donor site (exon 23)**
**c.9118-2A>G**	**BIC**	**Splice site**	**[-] Natural acceptor site(exon 24). [+] cryptic acceptor site 7-nt downstream**
**c.9148C>T**	**BIC**	**Nonsense**	**[+] 2 ESS**
**c.9172A>G**	**BIC**	**Missense**	**[-] SRp40**
**c.9177delA**	**BIC**	**Frameshift**	**[+] Internal acceptor site (NNSPLICE: 0,78)**
**c.9196C>G**	**BIC**	**Missense**	**[+] ESS (hnRNP-B)**
**c.9213G>T**	**BIC**	**Missense**	**[+] partially conserved ESEs, [+] ESS (hnRNP-H)**
**c.9218A>G**	**BIC**	**Missense**	**[-] ESE, [-] ESS; [+] ESS (hnRNP-B)**
**c.9227G>T**	**BIC**	**Missense**	**[+] 2 ESS**
**c.9248_9256+7del**	**BIC**	**Splice site**	**[-] Donor site (exon 24)**
**c.9252_9255delAACAinsTT**	**BIC**	**Frameshift**	**Donor site (exon 24): 0,95→0,46**
**c.9256G>T**	**BIC**	**Nonsense**	**[-] Donor site (exon 24)**
**c.9256+1G>A**	**BIC**	**Splice site**	**[-] Donor site (exon 24)**

Naturally occurring mutations are shown in bold type. Artificial mutations targeted to conserved motifs are shown in italics.

HGVS nomenclature: Human Genome Variation Society, (http://www.hgvs.org/mutnomen); **Source**: BIC, The Breast Cancer Core Database; Artificial, artificial variants that we generated to disrupt putative splicing motifs / **Bioinformatics analysis**: [-], disruption; [+], creation; EX, exon; nt, nucleotide; del, deletion; ESE, Exonic Splicing Enhancer; ESS, Exonic Splicing Silencer; SF2/ASF, SC35, SRp40 and SRp55 are SR proteins that bind ESE/enhancer motifs; hnRNP proteins bind ESS/silencer motifs.

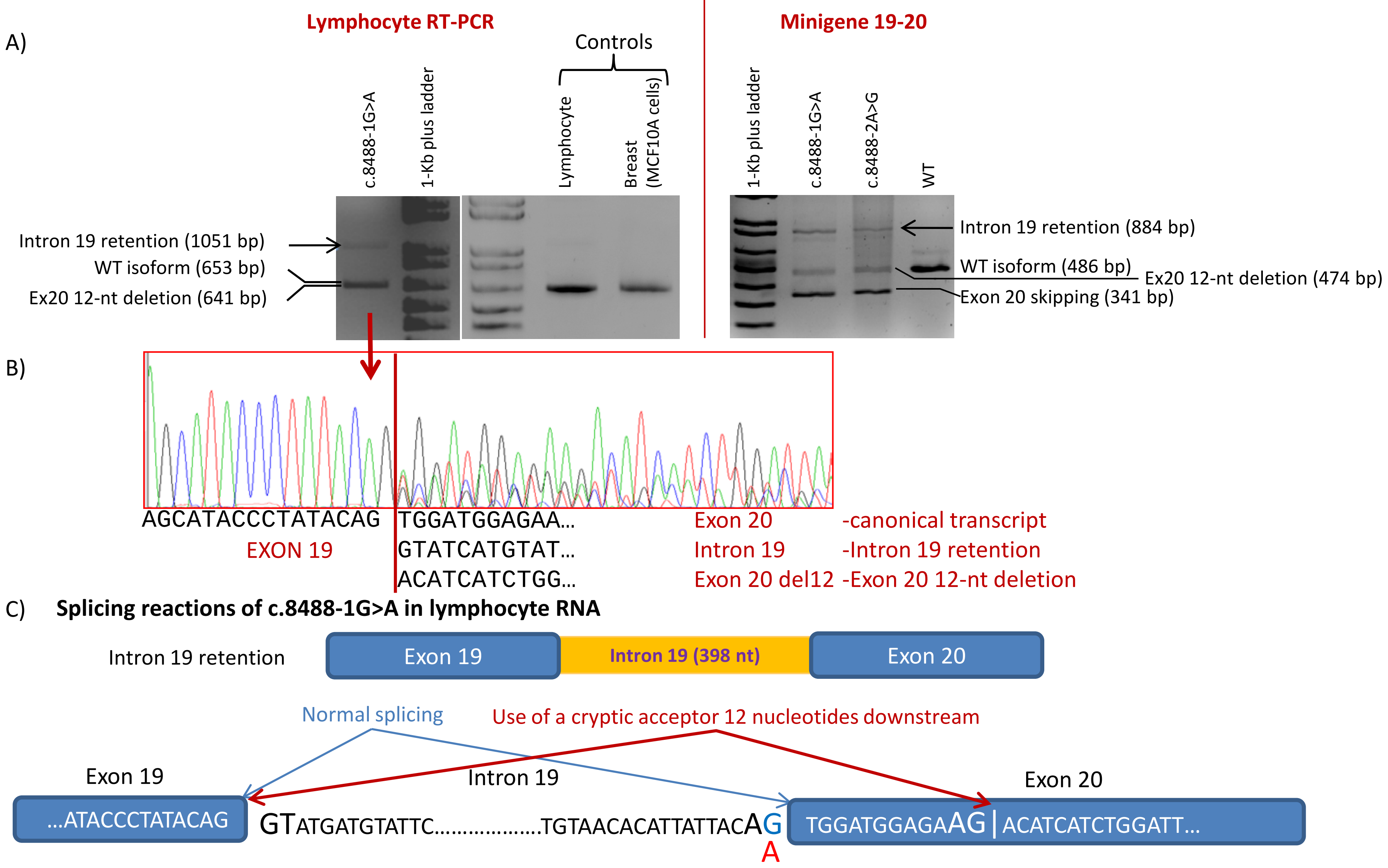

Figure S1. Splicing outcomes of DNA variant c.8488-1G>A. RT-PCR of lymphocyte RNA from patient harboring *BRCA2* c.8488-1G>A and controls (A-left) and minigene analysis of variants c.8488-1G>A and c.8488-2A>G that induced similar splicing outcomes (A-right). B) Sequence with primer RTBR2_EX19-FW of lymphocyte RT-PCR products induced by variant c.8488-1G>A. A mixture of three different sequences (vertical line) was observed downstream exon 19. C) Schematic representation of aberrant isoforms produced by mutation c.8488-1G>A. Mutation c.8488-1G>A induced intron 19 retention (indicated by an arrow) and a deletion of 12 nucleotides by the use of a cryptic acceptor site (see below) that could not be resolved from the wt band on agarose gels.

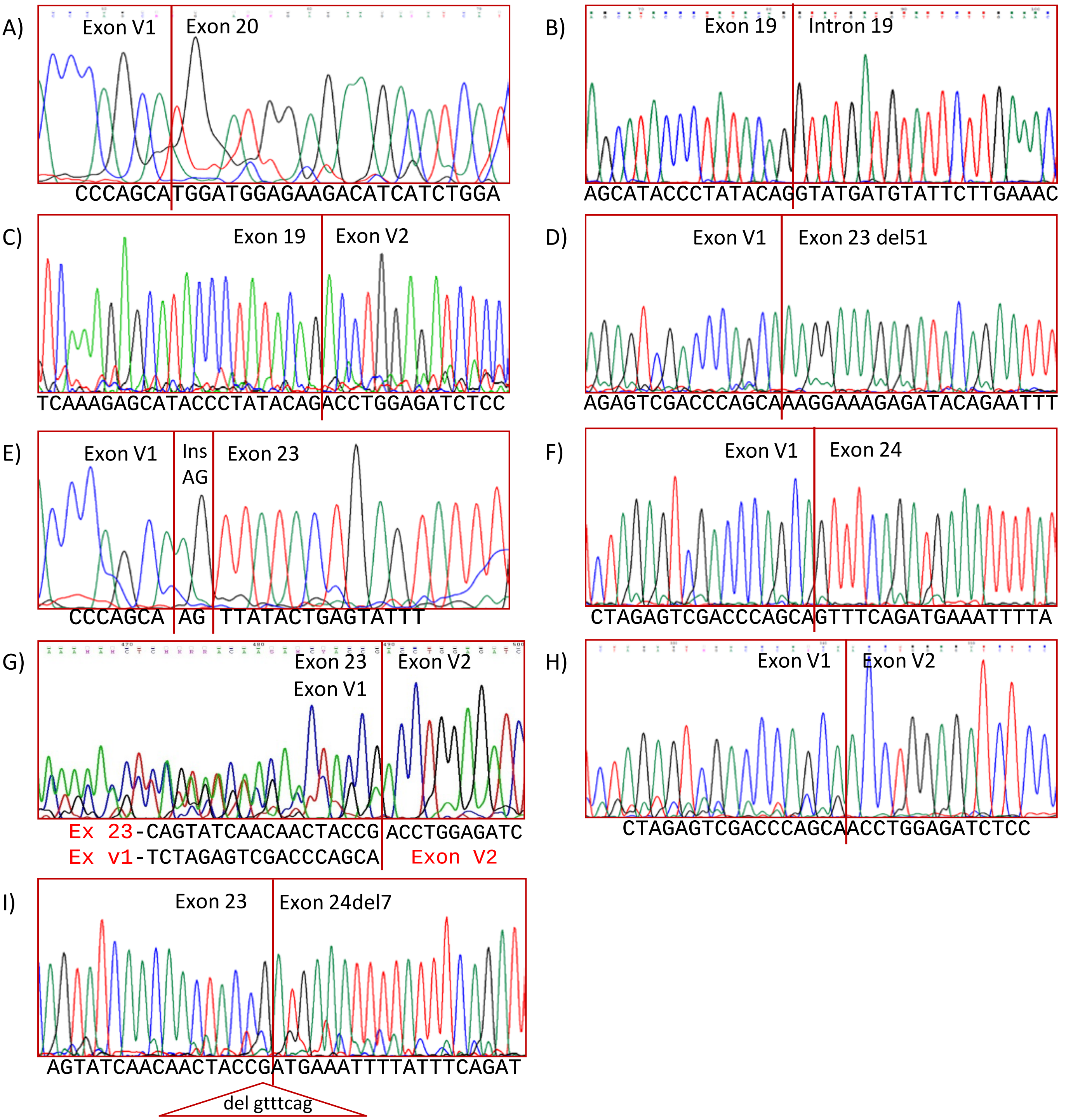

Figure S2. **Sequence chromatograms of the main splicing outcomes of the minigenes 19-20 and 23-24 of *BRCA2*.** V1 and V2 represent the vector exons. A) Exon 19 skipping (variant c.8486A>C); B) Intron 19 retention (c.8488-1G>A and c.8488-2A>G); C) Exon 20 skipping (c.8488-1G>A and c.8488-2A>G); D) Deletion of 51 nucleotides of the 5' end of exon 23 (c.8954-1_8955delinsAA); E) Insertion of 2 nucleotides in exon 23 (c.8954-3C>G); F) Exon 23 skipping (c.9117+1G>A); G) Exon 24 skipping and exons 23+24 skipping (c.9256+1G>A); H) Exons 23 and 24 skipping (c.9248_9256+7del); I) Deletion of 7 nucleotides of exon 24 (c.9118-2A>G). Three isoforms with deletions of the canonical transcript could not be sequenced. The first one was the 12-nucleotide deletion of exon 20 that was previously sequenced in lymphocytes (Supplemental Figure 1 B). The other two isoforms, exon 24del24 and exon 24del43, were the result of deletions of exon 24 whose sizes in capillary electrophoresis approximately matched with the possible activation of cryptic acceptor and donor sites, respectively, predicted by HSF (data not shown).

A)

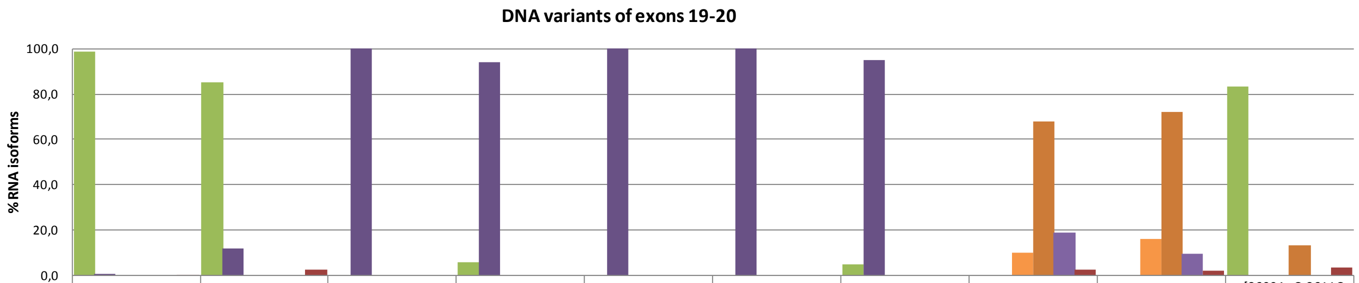

	WT	c.8378G>A	c.8486A>C	c.8486A>T	c.8487G>A	c.8487+1G>A	c.8487+3A>G	c.8488-2A>G	c.8488-1G>A	c.[8609A>G;8611G>T]
Canonical transcript	98,8	85,3		6,0			4,9			83,2
Ex 19 skipping	0,8	11,8	100,0	94,0	100,0	100,0	95,1			
Intron 19 retention								10,1	16,0	
Ex 20 skipping								68,0	72,1	13,2
Ex 20 12-nt del								19,1	9,7	0,0
Ex19+20 skipping	0,4	2,9						2,7	2,2	3,6

B)

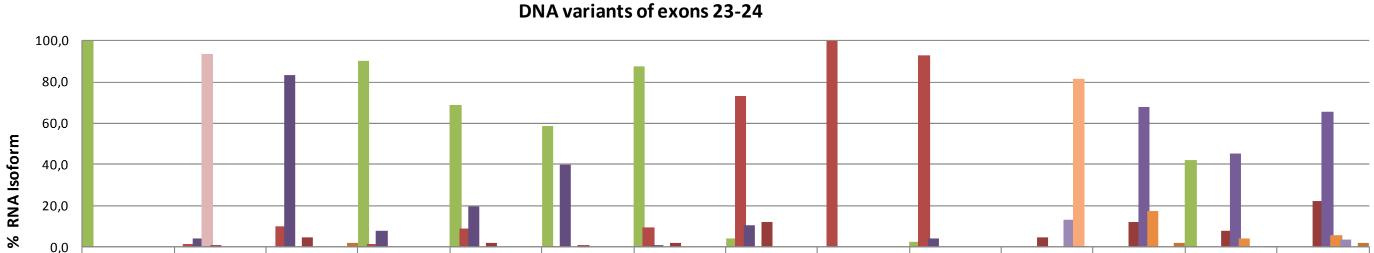

	WT	c.8954-3C>G	c.8954-1_8955delinsAA	c.8969G>A	c.9006A>T	c.[8972G>A;9006A>T]	c.9076C>T	c.9117G>A	c.9117+1G>T	c.9117+1G>A	c.9118-2A>G	c.9248_9256+7del	c.9256G>T	c.9256+1G>A
Canonical transcript	100,0			90,4	68,8	58,7	87,5	4,1		2,5			42,3	
Ex23 skipping		1,4	10,2	1,7	9,2	0,5	9,3	73,1	100,0	93,1				
Ex23del51		4,3	83,2	7,9	19,7	39,9	1,3	10,4		4,5				
Ex23 2-nt ins		93,5												
Ex23+24 skipping		0,8	4,5		2,3	0,9	1,9	12,4			4,9	12,5	8,0	22,6
Ex24 skipping												67,9	45,2	65,7
Ex24 43-nt del												17,5	4,3	5,8
Ex24 24-nt del											13,4			3,6
Ex24 7-nt del											81,6			
Other rare isoforms			2,1									2,1	0,1	2,2

Figure S3. **Quantification of RNA isoforms induced by variants of minigenes of exons 19-20 (A) and 23-24 (B)**. Semi-quantitative RT-PCR was performed in triplicate with a exonic pSPL3 FAM-primer. Samples were diluted in Hi-Di formamide (Applied Biosystems) and run in an ABI3130 Genetic Analyzer. Mean peak areas of three independent experiments were used to calculate percentage of each RNA isoform.

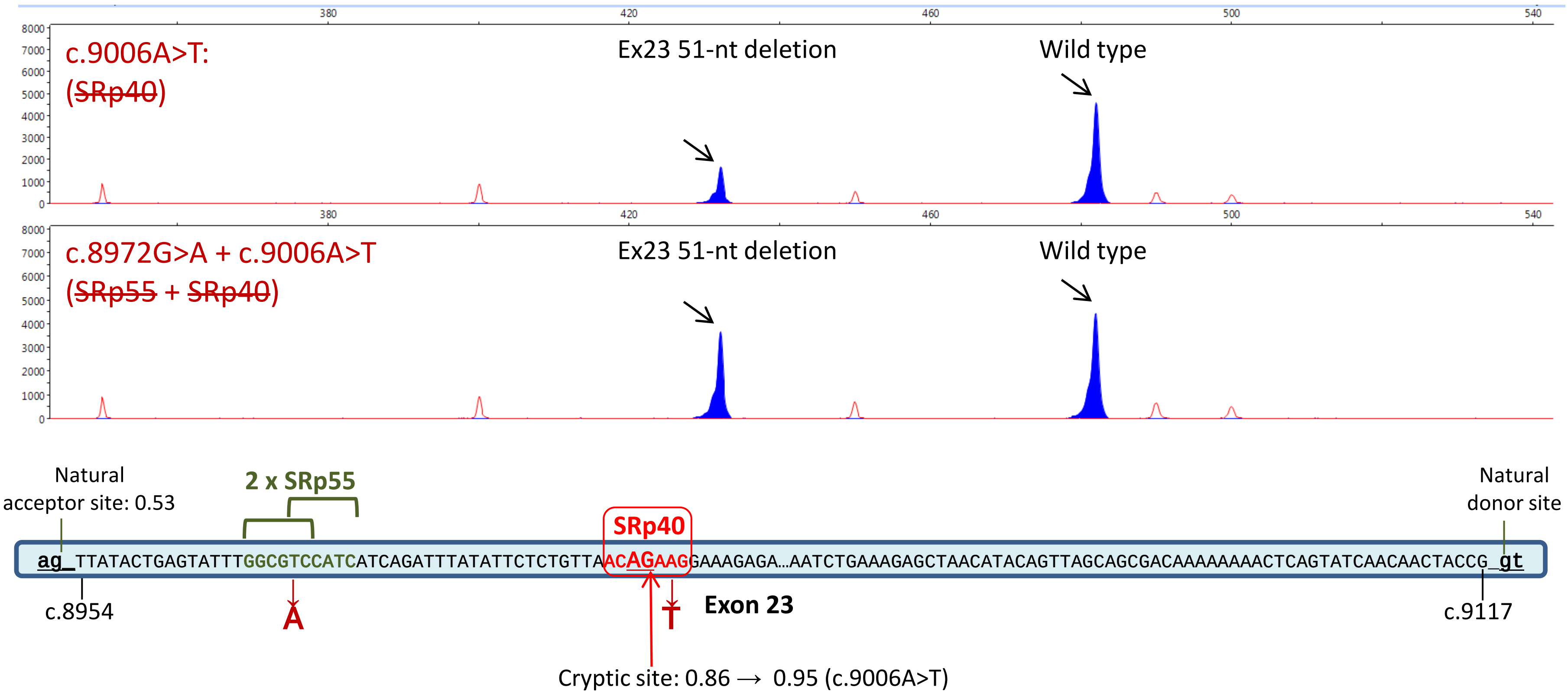

Figure S4. Cumulative effect of ESE mutations from BRCA2 exon 23 on splicing. Electropherograms of fluorescent RT-PCR assays of variant mutations c.9006A>T and double mutation c.9006A>T+c.8972G>A. Double mutant minigene was generated by PCR mutagenesis of c.8972G>A over the c.9006A>T minigene. Natural and cryptic acceptor and donor sites are indicated with the their NNSPLICE scores. The combination of both mutations increase the fraction of the aberrant 51-nucleotide deletion transcript (use of the cryptic splice site) from 19.7% to 39.9%.
